# Bridging global standards and local needs: a realist perspective on glocalisation in medical education

**DOI:** 10.3389/fmed.2026.1967360

**Published:** 2026-10-12

**Authors:** Ronan Daly, Muirne Spooner

**Affiliations:** 1 Royal College of Surgeons in Ireland, Department of Obstetrics & Gynaecology, RCSI Unit, Rotunda Hospital, Dublin, Ireland; 2 Royal College of Surgeons in Ireland, Department of Medicine, RCSI Education & Research Centre, Beaumont Hospital, Dublin, Ireland

**Keywords:** critical reflexivity, globalisation, glocalisation, medical education, post-colonialism, realist research

## Abstract

Contemporary interest in glocalisation partly reflects the limitations of earlier globalising movements in medical education. The former evolves to a process through which globally-informed educational models are negotiated and reconstituted within local contexts. While global standards can enhance graduate mobility, implementation may privilege Western assumptions regarding competence, professionalism, and quality. This can lead to obscuration of local values, needs and realities. Existing scholarship has examined glocalisation through sociocultural and postcolonial perspectives, generating insights into issues of hierarchy, power, and culture. Less explored is how methodology can empower transferable explanations of how global educational interventions generate different outcomes across contexts. We argue glocalisation can be considered a wicked problem: a complex, context-dependent phenomenon shaped by interacting pedagogical, regulatory, and socio-political forces. Methodological approaches capable of leaning into such complexity have potential in expanding knowledge regarding not only whether educational interventions succeed, but how, for whom, and under what conditions. We propose a critically-reflexive realist approach as a framework for advancing research and practice in this field. Realist inquiry enables researchers to identify how local actors interpret and enact globally-derived models within institutional, cultural, and material contexts. Such explanatory theories can generate actionable guidance for design and evaluation of glocalised educational interventions, while avoiding assumptions of universal applicability. Realist approaches must engage critically with power and representation to avoid reproducing colonial biases. We contend a critically-reflexive realist perspective offers a bridge between global standards and local needs, supporting more equitable, context-sensitive, and theoretically-informed approaches to medical education in an increasingly interconnected world.

## Introduction

Health professional global migration, global accreditation and regulatory practices, and transnational medical curricula are growing phenomena in medicine (1). One influential pattern involved curricula, pedagogies, competency frameworks and standards—often developed within Western institutions—being transferred across borders with insufficient attention to local educational traditions, health-system priorities and values (2, 3).

Glocalisation aims to conceptually marry the growing need for health professions education to be both internationally informed while remaining locally relevant and respectful (4). Global standardisation, comparability and regulatory assurance are legitimate objectives to promote mobility of graduates, diffusion of best practices, and reassurance regarding quality of medical education (2), necessitating dynamic negotiation with local systems and practices (5).

There is perhaps an inevitable tension therefore in developing a shared mental model of what glocalisation can and should be in health professions programmes. Medical curricula are not culturally neutral commodities which can be transplanted across contexts without consideration of the local culture realities (6). Emerging evidence suggests that inclusion within both academic and clinical learning environments is experienced unevenly by healthcare students, indicating that local adaption must also attend to equity, belonging, and participation, rather than culture alone (7). The dominance of Western paradigms in international medical education and publishing has shaped “global” curricula discussions (3), embedding perceptions and assumptions regarding quality (8), power dynamics (9) and ideologies (10). Western-oriented curricula present implicit tenets that are challenging to reconcile with the cultural normal and beliefs of non-western students, in areas ranging from professionalism (9) to professional identity (11). Local service provision needs may mismatch with global curricular requirements (11–14). Rigid application of training and assessment standards—often derived from Western educational traditions—may marginalise or obscure knowledge, practices and professional values that are locally meaningful. One example of this disparity is refugee and asylum seeker health, where increasing global migration has created locally significant healthcare needs that remain inconsistently represented in undergraduate medical curricula despite international recognition of their importance (15, 16).

Glocalisation offers far-reaching potential in leveraging shared knowledge and experience, developing culturally responsive curricula that produce healthcare practitioners with global-facing skillsets. Simultaneously, it throws up considerations—programme fidelity grapples with local imperatives; cross-border regulatory, operational disparities arise; ideological mismatches are common (14). This paper situates glocalisation, therefore, as a “wicked problem”.

## Addressing glocalisation as a wicked problem

We suggest that research into glocalisation warrants consideration of the concept as a wicked problem. Wicked problems are issues which are context-dependent, unpredictable, open to external influences, and involve many aspects and considerations (17, 18). Negotiation and enactment of global with local priorities in health professions education presents challenges. Stakeholders may disagree on what glocalisation aims to achieve and what implementation and success look like. Objectives may conflict, for example, regulators favouring equivalence, institutions international reputation, learners’ professional mobility, local communities how graduates support health needs. There is no context-independent conceptualisation of “perfect” glocalisation. In order to address this complexity, any model adopted for research into glocalisation must be capable of processing such a wicked problem.

## How glocalisation has been conceptualised and researched

Glocalisation has only relatively recently gained prominence as an explicit organising concept in HPE. Many of its central concerns have long been examined through scholarship on culture, cross-cultural learning, internationalisation and transnational education. Both theoretical and methodological approaches to glocalisation are therefore heterogeneous, reflecting the diversity of phenomena, contexts and research purposes in the field. Theoretical and conceptual lenses have included cultural frameworks, sociocultural and system theories, and post-colonialism. While Hofstede's cultural dimensions (19) have been used in research to frame national cultural differences across values such as communication (20, 21), hierarchy (22) and collectivism (4, 23), the model has been criticised as assigning a systematically causal status to culture and marginalising other factors in the attribution of causation to complex outcomes (24). Indeed, critics argue that his conceptualisation of culture as nationally homogenous is artificially bounded (25) and cannot account for intersectionality with small cultures (26) and personal identity (27). Sociocultural learning theory (28) and Figured Worlds (29) provide lenses through which to explore phenomena frequently encountered in glocalisation literature such as power, identity and cultural norms (30, 31). Frambach et al. (2014) noted that activity theory, borne out of socio-cultural learning theory, as its central dictum considers that all learning occurs through activities, providing a framework by which to analyse complexity of interactions between an individual and an environment (32).

Beyond theory, it is further illuminating to consider some conceptual positions assumed within glocalisation work, much of which have focused on the directionality and power relations of educational transfer. Acknowledging a need to move beyond a Western transplant model, postcolonial and decolonial perspectives therefore foreground reflexivity and ask whose knowledge defines quality, whose priorities determine what is adapted, and who retains control over educational resources and standards. Neo-colonial assumptions may persist where local stakeholders are consulted but globally powerful actors continue to define the problem, legitimate forms of knowledge and acceptable outcomes (33, 34). At a more specific conceptual level, Frambach et al. (2019) explored PBL in healthcare education, highlighting two dominant global discourses in the field: universalism and culturalism, the former aligning ideologically with uniform implementation, the latter privileging adaption to local contexts and values with their alternative conceptualisation of “plural PBL” acknowledging multiple, locally constituted practices shaped by the actors, relationships and systems through which it is enacted (35).

Methodological diversity has also characterised glocalisation research, delineating its inherent complexity and the multitude of scholarship focusses it inspires. Comparative case studies and document analyses have examined how international accreditation standards are adapted within national systems (4) while multi-site cross-cultural qualitative studies have explored how globally mobile pedagogies and educational practices are interpreted and experienced across settings (31, 36, 37). Quantitative cross-national research has compared cultural dimensions among medical learners (38) and examined associations between national cultural characteristics and curricular adoption (39). Longitudinal mixed-methods research has also evaluated how international medical programmes prepare graduates for cross-border practice (40). These studies have collectively generated important accounts of contextual variation, stakeholder experience and processes of adaptation and negotiation.

The methodological approach adopted shapes not only how glocalisation is investigated, but also which aspects of it become visible and what kinds of knowledge can be produced. Table 1 uses the implementation of internationally derived accreditation standards within glocalised HPE programmes to illustrate how different purposes of inquiry shape research questions, engagement with theory, methodological choices and the forms of knowledge produced. The approaches are presented as a heuristic rather than as mutually exclusive or hierarchically ordered alternatives, and illustrating the distinctive contribution of realist inquiry lies not in its use of particular data sources, but in its explicit development and refinement of contingent causal explanations.

**Table 1:** Illustrative example of methodological approaches to a focus of inquiry in glocalisation. This table uses the study of international accreditation standards in glocalised HPE programmes to exemplify how research focus impacts methodological choices.

Methodological approach	Principal focus	Illustrative research question	Potential data sources	Potential role of theory conceptual framing	Analytic approach	Knowledge generated
Phenomenology	The meaning and lived experience of a phenomenon	What is the lived experience of educators negotiating international accreditation standards with local educational and health-system priorities?	In-depth interviews, reflective accounts and, depending on the phenomenological tradition, observations	Interpretive or critical phenomenology may draw on postcolonial or decolonial perspectives to consider how power, standardisation and marginalisation are experienced.	Descriptive or interpretive phenomenological analysis of participants’ experiential accounts	A rich account of what negotiating global standards and local priorities means to those experiencing it
Phenomenography	Variation in how people understand or experience a phenomenon	What are the qualitatively different ways in which students and regulators and educators understand the relationship between international accreditation standards and local needs?	Interviews designed to elicit variation in participants’ understandings	Concepts such as universalism and culturalism could be sensitising concepts in guiding the interview design	Development of categories of description and an “outcome space” showing their relationships	A structured account of the different ways the phenomenon is understood across participants
Constructivist grounded theory	The social processes through which action and meaning develop	How do educators and regulators adapt and institutionalise international accreditation standards over time?	Iteratively collected interviews, observations and documents, guided by theoretical sampling	Concepts from sociocultural, figured-worlds or postcolonial scholarship could serve as sensitising concepts to be examined in analysis development iteratively, if relevant.	Constant comparison, coding, memo-writing and progressive theoretical development	A substantive theory explaining the process through which standards are negotiated and adapted
Comparative case study	How a phenomenon is configured and unfolds across bounded settings	How and why does the adaptation of the same international accreditation standards differ across institutions or countries?	Interviews, observations, policy and curriculum documents, institutional records and quantitative indicators	If a lens prioritsiing how power and epistemic authority are constructed, Postcolonial or decolonial perspectives may be useful framing. Universalist, culturalist or plural positions could frame interpretations of fidelity and adaptation.	Within-case analysis followed by cross-case comparison, pattern matching or explanation building	Contextually rich comparison identifying similarities, differences and potentially influential conditions
Realist inquiry	How contexts interact with underlying causal processes to generate outcomes	What intended and unintended outcomes arise when internationally derived accreditation standards are implemented within local educational and health-system contexts?	Existing research and theory, policy documents, stakeholder interviews, observations and relevant quantitative outcome data	Existing theories and conceptual positions are deliberately used to inform initial programme theories, which are tested in analysis, and adapted or refined based on their explanatory contribution.	Retroductive and abductive analysis; development, testing and refinement of context–mechanism–outcome configurations and programme theories	Contingent causal explanations of how stakeholders respond to particular resources or opportunities under different conditions, supporting transferability through explanation

Thus, glocalisation research frequently focuses on differences and adaptions, less often developing causal explanations of how conditions alter what resources mean to students and what consequences follow. This situates realist inquiry as a potential approach in answering calls for transferable research into glocalisation that incorporates how phenomena behave differently in a diverse range of contexts (2, 10).

## A realist approach

We propose that realist inquiry may be one useful methodology in studying glocalisation. Sitting between constructivism and positivism, realism assumes that social phenomena exist in a reality independent of individual perception, and recognises that this reality can only be approximately known through human experiences, language, and culture (41, 42). In contrast to positivism, realism focuses on ontological, rather than epistemological, theories and conceptualises reality as a stratified open system in which unobservable factors interplay to produce observable changes (43, 44). In recognising this, realism is concerned with the causative and transformative interaction between agency, structure and relations (45). While multiple theoretical approaches can and have captured some of the complexity of this area, realism offers potential to also generate explanatory and actionable theories for practice.

Realism is a theory-driven approach which seeks to develop and advance understanding of how a complex intervention works, for whom, and under what circumstances (46). This conceptual framework has been used to address critical issues such as balancing health care professional education and patient care delivery (47), public involvement in healthcare research (48), and mental health education for health profession students (49). Realist inquiry seeks to understand interventions through generative, rather than successionist causation, illuminating how and why a programme succeeded or failed, not merely if it did so (45). Complex interventions require such complex explanations of causality to generate actionable and transferable guidance and insight (50). Realist evaluation enables researchers to identify patterns of interactions through context-mechanism-outcome configurations, describing how contextual factors interact and generate various outcomes through triggering or inhibition of particular mechanisms (51).

Realist inquiry centres on interrogating evidence to describe contexts that affect an intervention and the mechanisms by which they lead to an outcome, successful or otherwise. Contexts in realism refer to the individuals, interpersonal relationships, institutions and infrastructure which are involved and dynamically intersect in a programme (42). Such contextual factors impact the outcomes of a social system through mechanisms. Mechanisms are processes operating within an intervention that illustrate the way in which available resources are used. Mechanisms generate outcomes (either intended or unintended) and are activated or stymied by their context (52). Thus, the initial process in realist inquiry requires generation programme theories built on the context-mechanism-outcome configuration.

Wicked problems have innumerable causes, involving multiple actors, environments and frequently contesting ill-defined outcomes (53). Proposed interventions to address wicked problems often fail due to their innate complexity and the dynamic societal, structural and systematic forces at work (54). Wicked problems are often embedded within and symptomatic of other problems (17), resulting in difficulty identifying the point at which the problem can be determined to be “solved” (54). Realism has been put forward as a suitable methodological approach to investigate and address wicked problems, putting focus on evaluating the impact of multi-layered social systems and their interwoven chains of causality (45). Realism approaches complexity analytically, recognising that programmes are embedded in social systems and using explicit context-mechanism-outcome theorisation to direct inquiry, focus on regularities and identify interventional outcomes (42). Realism necessitates a multi-layered social reality in which interventions are carried out, including the individual, the interpersonal, the institutional and the infrastructural (51).

Applied to glocalisation, realist inquiry has the potential to reframe an often-reproduced conceptualisation of a fixed global intervention copy and pasted to a passive local context. What is understood as “global” in this situation generally refers to the original setting, which may actually be limited to an institution or even a programme, but has acquired wider authority through societal, political and scholarly networks, often Western-dominated. This positions knowledge produced elsewhere as at the “local” level, or of limited transferability. Realist inquiry can examine knowledge, practices, priorities generated at all settings, consider how these interact and how different actors within these contexts understand, challenge or reconfigure the, In this way, “local” knowledge is reified not just as a context to “test” or modify an externally-derived model, but as part of the initial information defining the intervention, challenging assumptions about mechanisms and outcomes and therefore shaping the wider programme theory. This is not to say programme theories developed cross-contextual are immune to challenge. This paradigm rather emphasises the inherent recursive and multidirectional nature of transferability, whereby explanations are open to ongoing production in ever-changing contexts, contributing to an expanding global knowledge bank.

Rather than defining a universally successful form of glocalisation, it would aim to develop transferable explanations of how particular approaches operate, for whom and under what circumstances. This is consistent with plural PBL's rejection of replication and standardisation (51), while realist inquiry adds an explicit focus on explaining how different outcomes are generated. For example, implementing refugee and asylum seeker health teaching within a UK medical curriculum may involve globally informed competencies related to migration health, but knowledge contributed by asylum-seeker and refugee organisations, communities, healthcare services, learners and educators may alter which needs, resources and outcomes define the curriculum itself. Realist inquiry could examine how these different forms of knowledge interact with local clinical exposure, faculty expertise, institutional commitment, and community partnerships to shape learning, thereby potentially informing wider migrant health curricula (16). An initial programme theory regarding refugee and asylum seeker health education could be developed through a review of the literature and partnership with key stakeholders such as migrant patients. Further stakeholder engagement with healthcare service providers, educators and students could add to this initial theory, it is quite conceivable that these groups will disagree on points—they may contest curricular priorities, learning activities, the outcomes to be achieved. This initial programme theory could then be tested and refined through realist interviews with stakeholders representing local community, educational, institutional, and global perspectives, allowing for the synthesis of experiential, professional, organisational, and policy-level knowledge into a more robust explanatory account of outcomes. Realist inquiry will not “solve” the wickedness of the problem, but it will allow a deep focus on explanatory causal mechanisms, that lend themselves to testing and refining in differing contexts.

An example initial theory based on existing literature and stakeholder engagement may say that when migrant health is a part of a medical curriculum (context), students gain exposure to relevant cases and reflect on them (mechanism), leading to increased student confidence in caring for migrants as patients (outcome) (55, 56). As can be seen, such an initial theory takes a particular lens focusing on mechanisms and outcomes that are learner-centred leaner outcomes. A realist approach will test such a theory, examining curricular documents, interviewing students, educators, patients, community partners, seeking confirming and disconfirming evidence. This could provide a more contextualised understanding of both intended and unintended mechanisms and outcomes through the development and refinement of this theory. Drawing on multiple stakeholder perspectives could elucidate specific contexts such as the othering of migrant health narratives (57), the curricular marginalisation of asylum health education (58), and programme alignment to local needs and accreditation standards (59). Students who experience migrant health as a core, assessed curricular element delivered in partnership with community members may consider it clinically relevant; while those who have an isolated teaching may devalue it and this actually reinforces othering or tokenism. The refined theory would therefore consider broader contexts such as institutional legitimisation and how this is operationalised; more complex mechanisms with multiple actors and more practice-relevant outcomes. This theory can then be further tested in other settings to see what parts hold and which require refining in that context.

In adopting a realist approach with stakeholder input, the wicked problem of migrant health implementation can facilitate explanation of be more clearly defined, enabling the identification of the contextual factors and underlying mechanisms that shape outcomes across diverse settings, and supporting the development of responsive, effective and context-sensitive interventions.

Research relevant to glocalisation serves diverse purposes such as illuminating lived experience and understanding situated meaning and variation across contexts. What has been less developed in the literature are explicit, contingent explanations of how contextual conditions shape stakeholder reasoning and responses and how these contribute to differing mechanisms and subsequently differing outcomes. For questions of this kind, realist inquiry offers a particular form of explanatory transferability through the development and refinement of programme theories (60). It does not assume that an intervention, finding or model can be transferred unchanged between settings (61). It seeks to explain how particular processes generate outcomes under particular conditions, producing theories that can subsequently be examined and refined elsewhere. Realist inquiry may therefore be particularly suited to those areas of glocalisation scholarship concerned with how, why, for whom and under what conditions different intended and unintended outcomes arise. Developing such explanations encourages both methodological and theoretical pluralism.

Initial programme theories may draw on existing empirical literature, qualitative and quantitative evidence, policy and curricular documents, stakeholder knowledge and relevant substantive theories, before being iteratively tested and refined through primary research (52). Existing theoretical frameworks can provide sensitising concepts and candidate explanations without determining in advance what the inquiry will find. For example, Kehoe et al. drew on Kirwan and Birchall's transfer-of-learning model to conceptualise individual, training and organisational influences on international medical graduate transition, while Harrison and Shaffer's Adjustment–Effort–Performance Model informed their conceptualisation of transitional outcomes (62). Realist inquiry therefore calls for methodological and theoretical agility: researchers must move recursively between different forms of evidence, stakeholder accounts and potentially complementary frameworks while maintaining an explicit and coherent programme theory. Such pluralism requires restraint. Researchers must remain critically attentive to what each theory contributes, the assumptions and potential biases it carries, and points of compatibility or tension between frameworks. The explanatory value of each theory should be revisited as programme theories are developed, tested and refined, rather than treating any framework as fixed or universally applicable.

Realist review and primary realist inquiry may also operate as complementary phases within a wider programme of theory development. A realist review functions beyond aforementioned data sources; it is a theory-building form of evidence synthesis through which provisional programme theories are developed, tested and refined against evidence from multiple settings. These review-derived explanations can subsequently be interrogated through primary inquiry in particular institutional, cultural or regulatory contexts. The process is iterative rather than confirmatory: primary data may support existing configurations, but may also differentiate, challenge or extend them.

This recursive movement between cross-contextual synthesis and situated inquiry is particularly relevant to glocalisation. A realist review can identify explanatory patterns that recur across countries and institutions, while locally situated research can examine how those explanations travel, which contextual conditions they overlook and whether the outcomes they privilege are locally meaningful. Local findings therefore do not just provide a setting-specific application of globally derived knowledge; they can reconstitute the wider programme theory and contribute back to its transferability.

This can be explained further through our own work in the area of case-based learning, whereby initially a realist review was undertaken, we first developed two provisional programme theories informed by adult learning theory and inquiry-based learning theory, both commonly cited as theoretical foundations for CBL. The realist review then interrogated and elaborated these explanations against evidence synthesised from eligible studies. This extended the original work to seven programme theories, generating transferable and generalizable theories regarding case facilitation, institutional support and psychological safety in international CBL interventions (63). These review-derived theories then provided a starting point for a subsequent realist evaluation with students and faculty across two campuses of an international medical school delivering a shared CBL-centred curriculum. (Daly et al., manuscript submitted for publication).

## Critical reflexivity and realism in glocalisation

Reflexivity refers to the ongoing examination of how the positions, assumptions, theoretical commitments and institutional relationships shape the development and interpretation of programme theory (64). Reflexivity in realist research should involve the combination of realist methodological experience with the contextual, cultural and linguistic expertise from involved sites in the careful composition of research teams (65). Reflexive team discussions, attention to translation decisions, seeking disconfirming accounts and maintaining a programme-theory audit trail are examples of how reflexivity can be implemented in a practical sense (64). We highlight how reflexivity, while a cross-cutting methodological responsibility, is potentially of heightened importance in realist glocalisation research as hidden assumptions can become embedded in programme theories and subsequently appear to be neutral causal explanations. This is outlined in Table 2, which describes areas of potential obscuration linked to suggested reflexive practices.

**Table 2:** Illustrative example of reflexive approaches at different stages of realist inquiry to potential areas of obscuration in glocalisation.

Stage of realist inquiry	Potentially obscured risk in glocalisation research	Illustrative reflexive practice
Research-team formation and governance	Global lead institutions retain authority over questions, methods and interpretation, while local researchers primarily facilitate access or data collection	Team expertise should straddle methodology, cultural context, institutional knowledge and stakeholder expertise from each setting; agree decision-making, authorship and ownership arrangements
Defining the problem and outcomes	Internationally or institutionally determined objectives are treated as self-evidently desirable	Ask who defines the problem(s) under inquiry. Involve students, educators and relevant patient or public partners in identifying valued and unintended outcomes
Initial programme theory development	Initial theories may privilege data derived from WEIRD populations, marginalising local knowledge systems and	Critically examine inclusion and exclusion criteria. Consider theoretical influences and identify geographical, epistemological and linguistic origins. Seek guidance from local stakeholders before finalizing candidate theories.
Data collection & synthesis	Colonial histories, power relations, and marginalised perspectives may be overlooked, leading to incomplete understandings; ideological assumptions may shape data interpretation and theory generation.	Explicitly examine historical and sociopolitical influences; seek participant and local participation; maintain a reflexive research discussion and journal.
Interpretation, dissemination & knowledge mobilisation	Academic and Western priorities may dominate dissemination, limiting local ownership and benefit	Use collaborative interpretation, local-language dissemination, and reciprocal engagement with communities

Ensuring local researchers are involved with the development of research questions, theory development and interpretation of findings allows realist researchers to critically reflect on influence and power dynamics and bring situated knowledge to the fore (66). Student partnership and, where relevant, patient and public involvement could similarly help identify which outcomes matter and challenge assumptions within initial programme theories (49). In approaching glocalisation without critical reflexivity, initial programme theories may be disproportionately informed by the evidence and outcome definitions developed in Western or well-resourced settings. Similarly, the voices of key stakeholders in interventions are often minimised, due to factors such as translation issues and failures to appropriately address researcher-participant power dynamics which can lead to bias (65, 67). Mukumbang and van Wyke (2020) demonstrated how the use of technology such as Photovoice (incorporating self-captured images, interview and focus group data) provides researchers with the opportunity for participant-directed knowledge generation to inform theorising (68). Such approaches can foster a bidirectional flow of knowledge and influence in glocalisation. Adopting a critically-reflexive realist approach enables stronger attention to equity, enhancement of local agency and voices, and co-construction of knowledge between local and global stakeholders. Local actors can therefore be empowered to shape international discourse while ensuring locally relevant solutions and function both as adaptors of, and genuine contributors to, global knowledge.

## Limitations of realism

While we propose that realist inquiry may contribute to glocalisation scholarship by developing understanding of mechanisms and generating transferable explanations, its application has limitations. Operationalising multisite realist inquiry may require a team combining realist methodological experience with cultural, linguistic, institutional and stakeholder expertise from each setting. Although such expertise may exist locally, differences in available time, funding, research infrastructure and access to realist training can make these teams difficult to assemble, resource and coordinate equitably.

The theory-driven nature of realist inquiry also means that initial programme theories are shaped by the available scholarly landscape. The over-representation of Western, Educated, Industrialised, Rich and Democratic (WEIRD) perspectives in academic research may create an epistemic echo chamber in which Western assumptions about how interventions work, what outcomes are desirable and what constitutes success are reproduced within initial theories (51). Incorporating local scholarship, policy documents, diverse empirical evidence and stakeholder knowledge, followed by iterative testing across settings, can challenge these assumptions. However, reflexivity alone cannot fully compensate for perspectives that are absent from the evidence base. If insufficiently addressed, programme theories may be both explanatorily incomplete and limited in whose knowledge they represent, reducing their relevance and transferability across contexts.

## Conclusion

Glocalisation scholarship continues to challenge researchers in developing models of inquiry that seek to answer a range of underexplored areas. Glocalisation involved ongoing negotiation among educational models, people, systems and values. Realist inquiry has a focussed purpose to add knowledge about what works for whom and in what contexts it is particularly suited to bring tangible explanations to a phenomenon laden with complexity. Its value lies in supporting transferability not through universal prescription, but through explanation. Realising this potential requires methodological agility, equitable cross-contextual partnerships and critical reflexivity about whose knowledge defines both the intervention and its success. Used thoughtfully, realist inquiry can connect rich accounts of contextual difference with action that is locally relevant and globally informative.

## Data Availability

The original contributions presented in the study are included in the article/Supplementary Material, further inquiries can be directed to the corresponding author.
